# Evaluation of DNA in Human Teeth—Ante-Mortem and Post-Mortem Factors Affecting Degradation and Preservation: A Literature Review

**DOI:** 10.3390/genes16040364

**Published:** 2025-03-22

**Authors:** Ana María Salazar, Patricia Alejandra Huerta, Viviana Coliboro-Dannich, Ariel F. Castro, Anna Barbaro

**Affiliations:** 1 Department of Pathology and Diagnosis, Faculty of Dentistry, University of Concepción, Concepción 4070409, Chile; 2 Doctoral School of Forensic Sciences, University of Alcalá, 28801 Alcalá de Henares, Spain; 3 Department of Public Health, Faculty of Medicine, University of Concepción, Concepción 4070409, Chile; patriciaahuerta@udec.cl; 4 Department of Biochemistry and Molecular Biology, Faculty of Biological Sciences, University of Concepción, Concepción 4070409, Chile; vcoliboro@udec.cl (V.C.-D.); arcastro@udec.cl (A.F.C.); 5 Studio Indagini Mediche E Forensi (SIMEF), via Nicolò da Reggio 4, 89128 Reggio Calabria, Italy; 6 Instituto Universitario de Investigación en Ciencias Policiales, Universidad de Alcalá, Libreros 27, 28801 Alcalá de Henares, Spain

**Keywords:** DNA, teeth, degradation factors, identification

## Abstract

The identification of human remains is a major challenge in forensic science, particularly in cases of advanced decomposition. Human teeth are among the most resilient tissues to environmental and post-mortem degradation, making them a valuable source of DNA for forensic identification. However, DNA preservation in teeth is influenced by multiple factors that can compromise the success of genetic analysis. Objective: This review analyzes the intrinsic and extrinsic factors affecting DNA preservation in human teeth, focusing on ante-mortem and post-mortem variables rather than the methodological aspects of DNA extraction and analysis. Methodology: A review of the literature was conducted, evaluating studies that assess the impact of biological factors (such as dental structure, pathology, and treatment) and environmental conditions (such as burial environment, temperature, and humidity) on DNA degradation in human teeth. Results: The findings indicate that DNA preservation is significantly influenced by the type of tooth, the presence of dental restorations or pathological conditions, and exposure to environmental factors. While some studies address these variables individually, forensic cases often involve complex interactions among multiple factors, making DNA recovery outcomes unpredictable. Conclusions: The degradation of DNA in human teeth results from a multifactorial process where intrinsic and extrinsic elements interact dynamically. A comprehensive understanding of these factors is essential for optimizing sampling strategies and improving DNA recovery success rates in forensic applications. Future research should aim to develop predictive models that account for these variables, enabling more effective case-specific approaches to forensic DNA analysis.

## 1. Introduction

Following tragic events such as crimes, fires, mass disasters, terrorist attacks, and incidents involving weapons of mass destruction, the identification of human remains often relies on forensic DNA analysis, particularly when tissues are highly degraded or fragmented. In forensic contexts, human remains are frequently recovered in advanced stages of decomposition, exposed to extreme environmental conditions, or fragmented due to taphonomic processes or traumatic events. These include immersion, burial, prolonged exposure to open-air environments, fires, and fatalities resulting from asphyxia or drowning. In such cases, conventional soft tissue samples are often unsuitable for DNA analysis, making hard and mineralized tissues—such as teeth and bones—critical sources of genetic material. Due to their exceptional resistance to degradation and decomposition, these tissues significantly enhance the likelihood of successful DNA retrieval, playing a fundamental role in forensic identification [1,2].

Teeth are considered a valuable source of DNA. To understand this concept, it is necessary to examine the tissues that compose them. Teeth are located within the oral cavity and are protected by bone and muscle tissue, inserted within a bony socket called the alveolus. They are divided into two parts:

- The crown is exposed to the oral environment and composed of enamel, dentin, and pulp.
- The root is located within the bone tissue (alveolus) and composed of cementum, dentin, and pulp.

### Tooth Characteristics and Affecting Factors

It is well established that both bone and teeth are among the most resilient human tissues due to their high mineral content, primarily in the form of hydroxyapatite crystals (approximately 60% in bone tissue and 95% in dental enamel). This mineral composition facilitates the formation of bonds with DNA molecules, enhancing their resistance to degradation and decomposition. Consequently, both tissues are widely used as DNA sources in cases involving extreme degradation.

However, this review focuses specifically on human teeth, as they have been shown to be even more resistant than bone tissue. This increased resilience is primarily due to the protective role of enamel, which is significantly more mineralized than bone, shielding the DNA within the pulp and dentin. Additionally, the presence of cementum and alveolar bone surrounding the root provides further protection, while the intraoral location of teeth offers an added barrier against environmental degradation.

Although, histologically, bone contains a higher number of cells per unit area from which DNA can be extracted, several studies have reported that teeth yield better DNA recovery compared to bone. This observation is based on the intrinsic properties of dental tissues and post-mortem preservation factors rather than on variations in DNA extraction techniques and protocols.

Enamel is highly mineralized, being one of the hardest tissues in the human body due to its high mineral content. It is composed of 95% mineralized substance (calcium phosphate in the form of hydroxyapatite crystals), 1% organic matter, and 4% water [3]. Additionally, it is acellular, meaning it does not contain DNA. However, due to these characteristics, it forms a strong barrier against microorganisms, environmental, physical, and chemical effects, and external contaminants that could degrade DNA.

**Figure 1:**
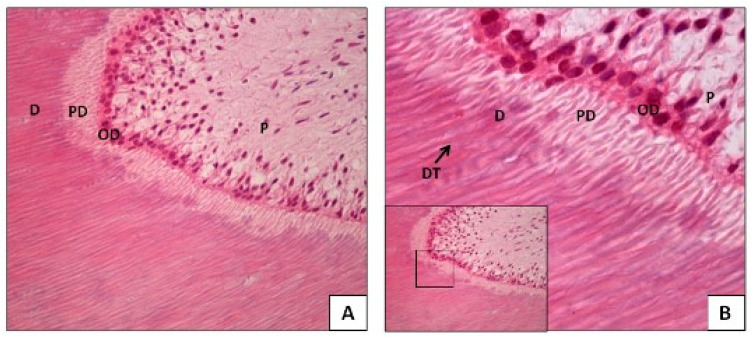
Histology of pulp–dentin complex (hematoxylin–eosin technique, optical microscopy, Faculty of Dentistry, University of Concepción): (**A**) 40×; (**B**) 100×. D: dentin, PD: predentin, OD: odontoblast, P: pulp, DT: dentinal tubules.

Conversely, pulp and dentin (the pulp–dentin complex) are highly cellular. The pulp tissue, located in the innermost part of the tooth, contains various cell types, mainly odontoblasts and fibroblasts, as well as immune cells. It is highly vascularized and innervated. Odontoblasts, situated on the periphery of the pulp adjacent to the dentin, have extensions that travel through tubules along the dentin (Figure 1). While these extensions do not contain a cell nucleus, they do contain mitochondria, making them a source of mitochondrial DNA (mtDNA). It is important to note that the pulp connects through the apical foramen and accessory canals to the cementum, the outermost layer surrounding the tooth root, which contains cells called cementocytes. It also connects to the surrounding periodontal tissues, such as the periodontal ligament, which contains fibroblasts, and the bone tissue. For this reason, some studies have successfully extracted DNA from endodontically treated teeth, where the pulp tissue has been completely removed, yet sufficient genetic material was still obtained for analysis. Due to their high cellularity and composition, the pulp–dentin complex and cementum are considered by many authors to be the dental tissues richest in genetic material [2,3,4,5,6,7,8,9,10,11].

Due to their morphology, location, and composition, teeth are considered the primary source of DNA in extreme cases. However, DNA extraction and analysis from teeth are not always successful, as various factors can accelerate, slow down, or halt the DNA degradation process. These factors can be classified based on whether their effects occur before death (factors inherent to the tooth and the individual, such as tooth type, age, and dental treatments) or after death (factors related to the environment and the time interval in which degradation occurs). Based on this, we distinguish between ante-mortem and post-mortem factors [12].

Although factors such as prior anthropological treatments, contamination risks, PCR inhibitors, and post-mortem molecular alterations can significantly influence DNA extraction outcomes, as this review focuses specifically on ante-mortem and post-mortem factors affecting human dental tissues and their impact on DNA integrity, we did not delve into aspects related to extraction techniques, methodologies, or contamination.

This review will analyze the ante-mortem and post-mortem factors described in the literature. The objective is to understand their impact on DNA degradation in teeth and optimize sampling strategies to increase the probability of success depending on the case at hand.

## 2. Methodology

The inclusion criteria for this review consisted of original full papers published in English and Spanish within the last 10 years, related to factors affecting DNA extraction from teeth. Additionally, related texts on the subject were included.

The following keywords were used: DNA, teeth, degradation factors, identification.

Studies that did not meet the language or time frame criteria, as well as those focusing on animal experimental models, were excluded. While recognizing the significance of non-human skeletal remains in forensic and archaeological contexts, this review specifically examines the environmental and biological factors influencing DNA preservation in human teeth, excluding other identification methods based on dental remains, such as age, height, and sex estimation.

### 2.1. Search Engines and Databases

A comprehensive search was conducted using databases such as MEDLINE/PubMed, Scopus, ScienceDirect, and others. To access Spanish research, platforms such as Dialnet, Redalyc, the Spanish National Research Council (CSIC), and RECyT were used, promoting open access to scholarly publications from Spanish universities and research institutions.

### 2.2. Search Methodology

An electronic search was conducted from January to December 2024, supplemented by manual searches. This approach aimed to identify studies that may not have been detected through electronic database searches.

## 3. Results

### 3.1. Main Ante-Mortem Factors

Ante-mortem factors are those that can affect the DNA of teeth before the individual’s death. These factors include the following:

**a. Type of Tooth:** Humans have different types of teeth—molars, premolars, canines, and incisors—each varying in size and morphology according to their function. During odontogenesis, the pulp tissue develops in shape and size, correlating with the number of roots and the crown morphology of each tooth. The literature suggests that a larger pulp volume provides a better DNA source [3,6,12,13]. Additionally, multi-rooted teeth, such as molars and some premolars, increase the success rate of DNA extraction. This is due to the presence of more cementum and multiple pulp canals, which contribute a greater number of cells and, consequently, more genetic material. Some authors [12,14,15,16] have even reported a higher proportion of DNA in the root of up to 10 times more than in the crown.

Furthermore, the posterior or distal location of molars and premolars in the oral cavity makes them less susceptible to environmental conditions or contaminants that could degrade DNA post-mortem [6,12]. Consequently, most published studies indicate that the preferred dental samples for DNA extraction are molars, followed by premolars [6,17,18,19].

However, some researchers argue that molars are the most affected by conditions such as cavities, restorative treatments, and endodontic procedures, which directly impact the pulp volume within the crown and root of the tooth [13,18,20,21]. On the other hand, some studies report no significant differences in DNA extraction success based on tooth type [6,12,22]. Therefore, the appropriate selection of a tooth for DNA extraction should be determined on a case-by-case basis;

**b. Biological Age of the Individual:** Since the pulp–dentin complex and cementum are the primary sources of DNA in teeth, another factor that could influence DNA extraction is the individual’s age at the time of death. This is due to morphological and histological changes that occur in these tissues, as well as in enamel, over time. For example, enamel becomes thinner, and secondary dentin deposition alters the overall structure of the tooth [23].

Odontoblasts, located at the periphery of the pulp in contact with dentin, secrete dentin matrix. Studies have shown that the number and secretory function of these cells deteriorate with age, directly affecting the quality and quantity of dentin [2,21,23,24,25]. These changes in both dentin and pulp primarily result from chemical, mechanical, physiological, or pathological stimuli experienced by the tooth throughout life. Examples include occlusal forces, gingival recession, hypersensitivity, and caries, which expose dentin to external stimuli, leading to the formation of reparative or tertiary (pathological) dentin and secondary (physiological) dentin [23].

**Figure 2:**
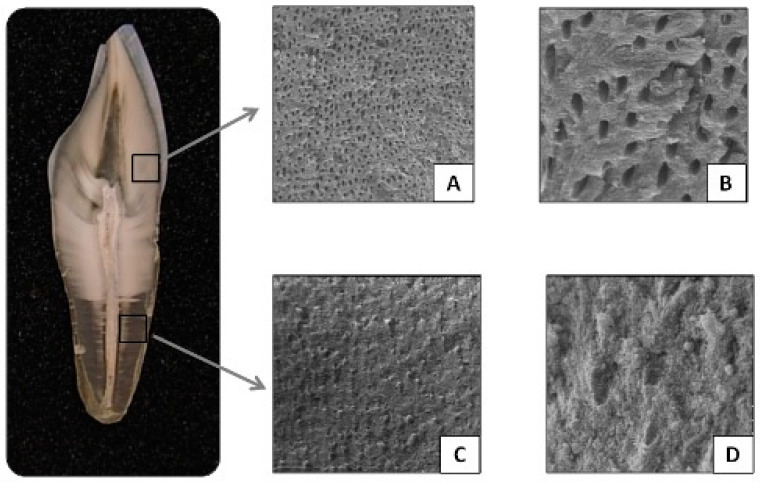
Root dentin transparency (SEM, Faculty of Medicine, University of Chile). Dentinal tubules in transverse section in physiological secondary dentin: (**A**) 500×; (**B**) 3000×. Occluded dentinal tubules in root sclerotic dentin giving origin to root dentin transparency: (**C**). 500×; (**D**) 3000×.

Therefore, significant differences have been observed in the diameter and number of dentinal tubules between young and older individuals. Younger individuals tend to have wider dentinal tubules, while in older individuals, these tubules become obliterated and mineralized, leading to physiological tubular sclerosis, which generally progresses from the root apex toward the crown (Figure 2). The increase in sclerotic dentin and the retraction of odontoblastic processes reduce the amount of viable tissue available for DNA extraction [23,26,27,28]. Additionally, secondary (physiological) dentin formation occurs at the dentin–pulp interface, further reducing the size of the pulp chamber [6,23,29]. The pulp volume in young individuals is significantly larger compared to that of older adults [23].

On the other hand, with age, pulp tissue undergoes cellular composition changes, becoming more fibrous and less cellular [27,29,30]. However, before tooth eruption, an acellular cementum layer forms over the root, but with age, there is a continuous deposition of cellular cementum. This increases the number of cementocytes available for analysis, while the greater thickness of cementum provides additional protection to the pulp against external microorganisms, potentially having a positive impact on DNA extraction [2,6,31,32].

**c. Pathological Changes in Teeth:** The previously mentioned changes that occur with age are physiological; however, pathological changes can also affect dental tissues. Severe pathological stimuli, such as dental caries, microbial infections, mechanical trauma (e.g., bruxism), or chemical erosion, can destroy the layer of odontoblasts in the pulp, leading to the rapid deposition of tertiary or reparative dentin. This type of dentin is tubular and reduces the volume of pulp tissue [21,27,29,31].

For this reason, dental pathologies must be considered when analyzing DNA from teeth, as the likelihood of pathological changes increases with age, leading to the loss of tissues that serve as sources for DNA extraction. Consequently, the success of DNA analysis may be affected. In this regard, Mansour H. et al. (2018) [33] state that when dentin is compromised by disease, the best source for DNA extraction is cementum, as it remains unaffected by these conditions. Their study concludes that even when a tooth has undergone severe ante- and post-mortem conditions, cementum preserves its morphological and cellular structure better than dentin, making it possible to obtain complete DNA profiles exclusively from cementum.

Another pathology that has not been fully studied is periodontal disease. This condition develops due to microbial invasion, leading to inflammation and infection of the periodontal tissues (gingiva, periodontal ligament, and surrounding bone). As a result, teeth lose their attachment to the bone, increasing mobility and ultimately leading to premature tooth loss. Contrary to expectations, Heathfield L. et al. (2021) [18] found in their research that a significantly greater quantity and higher quality of DNA could be extracted from these teeth. They suggest that this may be due to increased vascularization and the presence of blood cells in affected areas, resulting from inflammation and immune system activity. However, the extent of the disease must be considered, as advanced cases can expose cementum to the oral cavity, making it susceptible to cariogenic activity, which could destroy cementocytes [6].

The pulp–dentin complex is the primary source for DNA extraction in human teeth. However, in cases where access to these tissues is compromised, such as teeth with endodontic treatments, extensive caries, or prosthetic rehabilitations, alternative sources of genetic material must be considered. Among these, root cementum has been extensively studied, and recently, several authors have proposed that dental calculus could serve as a viable source for DNA extraction [34].

Dental calculus is a pathological mineralized deposit of bacterial plaque (biofilm)that adheres to the tooth surface, both supragingivally and subgingivally. This substrate contains microbial remains associated with periodontal disease (it is considered its etiological factor), food residues, and the individual’s endogenous microbiota. Due to these characteristics, it has been analyzed in multiple studies to assess dietary patterns, behavioral habits, and diseases in both contemporary and ancient populations [35,36].

In forensic science, dental calculus has been explored as a potential source of endogenous DNA due to its lower susceptibility to contamination and post-mortem degradation compared to conventional dental tissues. Its lower porosity, relative to dentin and cementum, has been suggested to provide greater resistance to environmental and temporal factors affecting DNA preservation after death [37,38]. However, the amount of DNA recovered from dental calculus is generally limited, and the precise mechanisms by which DNA becomes incorporated into this matrix remain unclear.

It is important to note that dental calculus is an external substrate of the tooth and is not present in all individuals. This implies that its analysis requires methodological approaches distinct from those used for conventional dental tissues. Therefore, its application in forensic and genetic studies should only be considered in cases where its presence is confirmed.

Based on this analysis, teeth selected for DNA extraction should ideally be free from diseases and dental treatments. However, such ideal conditions are not always present in identification cases, particularly in regions or countries where the prevalence of caries and periodontal disease is high. Additionally, differences in dietary habits compared to those analyzed in the existing literature may influence these factors.

**d. Composition and Mineralization of the Teeth:** The tissues with a high cellular content in the teeth (pulp–dentin complex and cement) are protected by mineralized tissues, such as bone and enamel, which are the tissues with the highest mineral content in humans. This characteristic makes these cells less susceptible to external post-mortem degradation factors. The mineralized collagen in bone tissue, enamel, dentin, and cement tends to be much more stable against these factors than the non-mineralized collagen in soft tissues, as this type of collagen in bone tissue and teeth forms calcium bonds with the apatite contained in them, causing the DNA macromolecule to become encapsulated within these [24,39,40,41].

Most of the inorganic mineralized component of the tooth is made up of calcium phosphate in the form of hydroxyapatite crystals, and various studies have shown that DNA has a high affinity for this molecule [42,43,44]. In the DNA structure, the bases are inside the double helix, and the sugar phosphates are on the outside. The phosphate groups carry a negative charge; therefore, DNA binds to hydroxyapatite due to the strong affinity between the phosphate groups and calcium ions. Okazaki M. et al. (2001) [44] reported that at a given pH, certain proteins bind to calcium sites on the surface of hydroxyapatite crystals through negatively charged acidic groups, while others, which are more basic, bind to phosphate groups on the surface. Based on this and the structural characteristics of DNA, hydroxyapatite tends to be more stable in alkaline solutions but becomes more susceptible to dissolution in acidic environments. This dissolution could expose DNA, making it more vulnerable to environmental degradation factors [43].

### 3.2. Main Post-Mortem Factors

Successful DNA recovery can also be influenced by environmental factors and the time elapsed since death, both of which contribute to post-mortem decomposition and degradation. Therefore, we classify post-mortem factors into the following categories:

**a. Environmental Factors:** In soft tissues, the post-mortem degradation process begins with the development of cellular autolysis, alongside the putrefaction of the tissues, where bacterial and fungal invasion occurs, leading to chemical degradation through the hydrolysis and oxidation of organic tissues, including the complete degradation of genetic material. This degradation process progresses over time and results in the breakdown of cellular organelles and DNA. Although the pulp–dentin complex and cement are well-protected against various degradation conditions, whether environmental or natural, they remain susceptible to these events [45]. It should be considered that degradation is affected by the nature and duration of this process and depends on the environmental conditions in which it occurs, such as exposure to moisture, temperature, ultraviolet light, substrate pH, and bacterial or fungal contamination. Depending on these conditions, the degradation process can be faster, slower, or even stop, meaning that the quantity and quality of the DNA obtained will also depend on these factors, which makes it difficult to predict [13,18,43].

*Temperature* is considered one of the main factors in the DNA degradation process [12]. The success of DNA extraction largely depends on the conditions to which the body has been exposed, with temperature playing a critical role. Low temperatures favor optimal DNA preservation for analysis by slowing most chemical reactions and reducing microbial colonization [46,47,48,49,50]. Conversely, high temperatures—such as those encountered in cases involving fire victims, explosions, or mass disasters—significantly alter the chemical structure of DNA, making identification more challenging. This degradation is directly proportional to temperature [12,43]. According to some authors [51,52], DNA extraction becomes increasingly difficult at temperatures above 400 °C as teeth become brittle and dentin undergoes structural deformation.

*Humidity* is a factor that negatively impacts DNA performance, as it allows organic substances and sediment contamination to penetrate the tooth, promoting the presence of inhibitor molecules and contamination with exogenous DNA [12,43,49,53,54]. Some studies suggest that DNA recovery is significantly more successful in dry environments than in humid ones (outdoor vs. indoor), which could mean that recovery is more successful in indoor, sheltered environments than outdoors [12,55,56,57]. However, the study by Mansour H. et al., 2019 [12] showed that DNA recovery from dental pieces in water is low but better than its performance in soil and humidity.

*Inmersion* is another negative factor for DNA extraction. This has been observed in different studies where dental pieces were submerged in seawater, showing a low success rate in performance [12,56,58]. According to García A. et al. [56], this could be due to the presence of microorganisms; however, the most probable explanation is the dilution effect of water, which would increase DNA hydrolysis [56]. However, other authors believe that the effect of seawater would not cause autolysis degradation of DNA [57,59,60].

The *pH* of the environment, including soil and water, plays a crucial role in DNA preservation. Hydroxyapatite, the primary mineral component of bones and teeth, exhibits lower solubility in neutral to slightly alkaline conditions, whereas acidic pH increases its dissolution. This dissolution accelerates the degradation of mineralized tissues, exposing DNA to external environmental factors that contribute to its deterioration [12,43,49,50,61,62,63]. Studies indicate that acid-induced demineralization increases porosity in bone tissue, allowing the infiltration of organic substances from the surrounding environment, thereby enhancing the susceptibility of DNA to contamination and degradation. A similar effect is observed in dental tissues, where demineralization of dentin and enamel facilitates the permeability of external substances through dentinal tubules, ultimately compromising DNA integrity [62,64]. Thus, maintaining the stability of the mineral matrix is essential for DNA preservation in mineralized tissues. The dissolution of hydroxyapatite in acidic environments exposes genetic material to degradation-promoting conditions, highlighting the importance of a neutral or slightly alkaline pH for the long-term preservation of both the mineral structure and associated DNA.

Soil composition also significantly influences DNA conservation. Certain compounds, such as tannins and humic acids, can enhance DNA preservation by inhibiting microbial degradation. Additionally, anaerobic conditions and high ionic strength in soil environments may contribute to DNA stability by limiting microbial and enzymatic activity [65].

The following outlines the primary soil types and their associated pH levels, emphasizing their impact on DNA preservation [66,67,68]:

1. Sandy soils: Characterized by a light texture and excellent drainage, sandy soils exhibit variable pH, ranging from slightly acidic to neutral. Their high permeability reduces microbial activity, which can favor DNA preservation;
2. Clayey soils: These soils possess a compact structure and high water retention capacity, often maintaining a neutral to slightly acidic pH. Their low oxygen permeability restricts microbial activity, thereby enhancing DNA conservation;
3. Silty soils: With an intermediate texture and good moisture retention, silty soils typically have a neutral pH, providing favorable conditions for DNA stability, as a neutral to slightly alkaline pH is optimal for genetic material preservation;
4. Calcareous soils: Rich in calcium carbonate, these soils maintain an alkaline pH, which inhibits microbial and enzymatic activity, promoting long-term DNA preservation;
5. Acidic soils: Soils with a pH below 7 can accelerate the decomposition of soft tissues due to increased microbial and enzymatic activity, leading to rapid DNA degradation.

The interplay between pH and soil composition is complex, and their combined effects on DNA preservation depend on site-specific environmental conditions. Studies have shown that humic acid-rich soils contribute to lowering pH, which, in turn, exacerbates DNA degradation [50,69]. Understanding the impact of these factors is essential for forensic investigations and archaeological research, as they directly influence the likelihood of recovering viable genetic material from human remains.

For all these reasons, it is also important to consider whether a body is found in indoor or outdoor conditions (indoor or outdoor), as the best results in DNA performance are obtained from samples recovered outside the reach of soil contaminants, UV light, and environmental water. Relatively dry environments, away from these environmental factors, prevent DNA damage [12,70];

**b. Post-mortem Interval:** Finally, the post-mortem time is another important factor in the DNA degradation process in dental tissues, and several studies consider the degradation process without taking this variable into account. Mansour H. et al., 2019 [12] specifies that post-mortem time is the most significant factor in DNA degradation, indicating that the most critical period for DNA preservation occurs in the first period after death, a finding that aligns with the results of other authors. The concentration and quality of DNA significantly decrease over time, with a notable difference observed in the median DNA concentrations [12,54,71]. These studies emphasize the importance of considering post-mortem time when analyzing DNA degradation in dental tissues, as DNA concentration decreases significantly in the first months after an individual’s death.

The importance of the post-mortem interval must be correlated with the environmental factors to which the samples were exposed. Despite being a highly relevant factor, it cannot be assessed in isolation. For this reason, the various analyses of this variable are controversial in the literature and difficult to evaluate.

The overall analysis of all reviewed aspects is summarized in Supplementary Table S1, which provides an overview of the main findings and trends identified in the scientific literature regarding DNA preservation in human teeth under various post-mortem conditions. This table highlights key factors influencing DNA recovery, offering a comprehensive perspective on how different environmental and taphonomic variables impact genetic material preservation.

## 4. Future Perspectives

The literature indicates that the most suitable dental pieces for DNA analysis are molars and premolars, provided they have no dental treatments or pathologies. The published studies use limited samples with these characteristics, but under real and natural death conditions, suitable samples are sometimes not available, especially in cases of crimes and mass disasters. For this reason, it would be of great importance to analyze samples that are not in ideal conditions, as this would allow us to consider different situations we might face in real cases and provide an adequate statistical sample size to establish parameters and an analysis guide for each case. It is worth noting that most of the available studies have been conducted on European, North American, or Asian populations, and very few studies have been conducted on South American populations. This opens new possibilities for analyzing the variables, as this population is exposed to different geographies, climates, customs, and dietary habits, in addition to having a different ancestral origin—characteristics that could affect these variables and would be interesting to analyze.

Finally, future studies should explore a direct comparison between teeth and bones under different environmental conditions, degradation stages, and forensic scenarios. Such research could provide valuable insights into optimizing DNA retrieval strategies and determining the most suitable tissue for genetic analysis in different forensic contexts. Additionally, a better understanding of how post-mortem factors differentially affect these tissues could further refine forensic methodologies and improve identification outcomes.

## 5. Conclusions

DNA analysis from human dental tissues has proven to be a fundamental tool in forensic identification, especially in cases where soft tissues are degraded or unavailable. The unique composition of teeth, particularly the pulp–dentin complex and cementum, makes them a valuable source of genetic material due to their resistance to adverse environmental factors.

However, the success of DNA extraction and analysis is influenced by multiple ante-mortem and post-mortem factors, which have been described in this review. Among the ante-mortem factors, tooth type, the biological age of the individual, and the presence of dental pathologies play a significant role in determining the quantity and quality of recoverable DNA. Post-mortem factors, such as environmental conditions, exposure to humidity, temperature, and soil pH, critically affect DNA preservation or degradation. Additionally, the evidence shows that, in most cases, the post-mortem interval is a key variable as DNA deterioration progresses over time, particularly in the initial months after death.

The reviewed studies highlight the complexity of DNA preservation in post-mortem teeth. While indoor environments seem to provide the most favorable conditions for long-term conservation, other factors—such as the time elapsed since death, exposure to moisture (especially saltwater), and high temperatures—have a significantly detrimental effect. The outcomes of burial vary and may depend on soil characteristics (pH and composition).

Tooth type appears to have a minor influence compared to environmental conditions, although some studies suggest differences in the amount of recovered DNA depending on the tooth type. The presence of dental pathologies may affect the success of DNA extraction, but the specific effects vary.

It is important to highlight that the existing scientific literature often focuses on one or a few specific variables, neglecting the fact that DNA degradation is a multifactorial process. Several researchers argue that a comprehensive and valid analysis should consider all variables together, as they are interdependent rather than independent. This limitation emphasizes the need for more extensive studies that examine the interaction of multiple factors simultaneously. Studies that fail to consider all relevant variables may lack the necessary validity. A deeper understanding of these processes would enhance extraction protocols and optimize genetic identification in complex forensic scenarios, particularly when working with human teeth, which serve as a critical source of DNA in numerous cases.

## Data Availability

No new data were created or analyzed in this study.

## Supplementary Materials

The following supporting information can be downloaded at: https://www.mdpi.com/article/10.3390/genes16040364/s1, Table S1: Summary Analysis of ante and post mortem factors in human teeth.
